# Concordance between appendicular skeletal muscle mass measured with DXA and estimated with mathematical models in middle-aged women

**DOI:** 10.1186/s40101-018-0179-5

**Published:** 2018-07-31

**Authors:** Nirmala Rathnayake, Gayani Alwis, Janaka Lenora, Sarath Lekamwasam

**Affiliations:** 1 Department of Nursing, Faculty of Allied Health Sciences, University of Ruhuna, Galle, Sri Lanka; 2 Department of Anatomy, Faculty of Medicine, University of Ruhuna, Galle, Sri Lanka; 3 Department of Physiology, Faculty of Medicine, University of Ruhuna, Galle, Sri Lanka; 4 Department of Medicine, Faculty of Medicine, University of Ruhuna, Galle, Sri Lanka

**Keywords:** Anthropometry models, Appendicular skeletal muscle mass, DXA, Women

## Abstract

**Background:**

Appendicular skeletal muscle mass (ASMM) is a measure of body muscle content, and it correlates with nutrition and physical status. Estimation of ASMM using anthropometric models is a well-established strategy to overcome issues related to the restricted availability of sophisticated techniques in measuring ASMM. This study aimed to assess the validity of four selected anthropometric models in estimating ASMM in middle-aged women in Sri Lanka.

**Methods:**

A group of women (*n* = 165) aged 30–60 years underwent a series of anthropometric measurements such as body weight, height, circumferences, and skin fold thickness at specific sites. The limb circumferences were corrected for subcutaneous adipose tissue thickness. Two models developed by Lee et al. (ASM 1, ASM2) and two models developed by Wen et al. (ASM3, ASM4) were validated using ASMM measured by dual energy x-ray absorptiometry (ASMM_DXA_) as the reference standard.

**Results:**

Mean (SD) age of the study group was 49.0 (8.2) years. Mean (SD) ASMM_DXA_ and ASMM estimated by the four models were ASMM_DXA_ = 15.39 (2.75) kg, ASM1 = 18.36 (3.27) kg, ASM2 = 16.46 (3.01) kg, ASM3 = 15.44 (2.40) kg, and ASM4 = 14.44 (2.45) kg. Correlations of ASMM_DXA_ with ASMM estimated by the models were as follows: ASM1, *r* = 0.68, *R*^2^ = 0.46, SEE = 2.02 kg; ASM2, *r* = 0.90, *R*^2^ = 0.81, SEE = 1.18 kg; ASM3, *r* = 0.90, *R*^2^ = 0.81, SEE = 1.17 kg; and ASM4, *r* = 0.91, *R*^2^ = 0.82, SEE = 1.14 kg. ASMM estimated by ASM3 was not significantly different (*P* > 0.05) from ASMM_DXA_ with mean difference of − 0.05 (range, 0.12 to − 0.23). Bland and Altman plot revealed satisfactory measurement agreements between ASM3 and ASMM_DXA_. The ASMM estimated by the other three models was significantly different from the ASMM_DXA_ (*P* < 0.05).

**Conclusion:**

The ASM3 model introduced by Wen et al. met all validation criteria and can be recommended for the estimation of ASMM in middle-aged women in Sri Lanka.

## Background

Skeletal muscle mass (SMM) is a key component of body composition, accounting for 30–40% of total body weight [1, 2]. It correlates with physical functions and health status [3] and involves in many processes related to health such as physiology, nutrition, clinical medicine [4], treatments, disease prevention, and long-term rehabilitation. Approximately 75% of SMM is located in the appendicular region [5] called appendicular skeletal muscle mass (ASMM), and reduction of ASMM leads to negative health consequences such as weakness, disability, impaired quality of life (QOL), and mortality resulting in increased health care burden [6, 7].

SMM is quantifiable using many techniques. Although standard techniques like MRI, CT, and dual-energy x-ray absorptiometry (DXA) are used to measure the SMM with high accuracy, they are expensive and not widely accessible and some have high radiation exposure. As an alternative, the indices of anthropometry, often in combination, are used to estimate or to predict SMM and the distinct advantages of anthropometry-based techniques are that they are noninvasive and inexpensive and can be applied in a wide range of clinical and research settings [8].

Martin et al. [9], in 1989, formulated a prediction model for SMM based on anthropometry with high precision (*R*^2^ = 0.93), and this model was further modified to make it more accurate and user friendly (*R*^2^ = 0.96) [10]. Later, similar studies had been carried out mostly in western communities [4, 11–14] and only a few have been reported from Asia [15, 16]. Of these studies, models introduced by Lee et al. [12] and Wen et al. [16] are applicable across a wide range of age while other models [4, 11, 13, 14] are more applicable for elderly population.

Lee et al. [12] introduced two formulae to estimate the ASMM in adults in the Western population comprising different ethnicities. They are based on anthropometric measures such as weight, height, circumferences, and skinfold thicknesses (SFTs) of selected sites, in combination with general information such as age, gender, and ethnicity. Wen et al. [16] too have adopted a similar approach for the Chinese population. Both models have been validated against the ASMM measured by MRI [12] and DXA [16] as the criterion method.

In Sri Lanka, the use of anthropometry in clinical decision-making is mostly limited to height, weight, and BMI with occasional use of abdominal girth. These are used as crude estimates of regional and global adiposity or when following guidelines based on these measures. Muscle mass, although shown to be a determinant of clinical outcome in many diseases, is hardly used in clinical decision-making in Sri Lanka due to lack of advanced technology to measure muscle mass and lack of guidelines for clinical applications.

It is pertinent to find methods that are inexpensive yet accurate and applicable in clinical settings to estimate ASMM for the use of clinicians and researchers. This would encourage research related to this area and subsequently in the development of guidelines for the use of muscle mass in clinical decision-making. The current study aimed at assessing the validity of selected mathematical models to estimate ASMM among middle-aged women. In order to allow for possible geographical variations in the predictors of muscle mass, we used four models, two validated among Western subjects and the other two validated in a group of Chinese subjects.

## Methods

### Study design, setting, and participants

This cross-sectional study was conducted in a group of 165 healthy community-dwelling women, aged 30–60 years randomly selected from permanent residents in the Galle District, Sri Lanka, during the period July 2015–July 2016.

Women who used medications that were likely to have an effect on muscle mass (thyroxin, corticosteroids, hormone replacement therapy (HRT), and oral contraceptives), who were on dedicated dietary or exercise programs, and with non-communicable diseases (NCD), polycystic ovarian syndrome (PCOS), and chronic pulmonary, cardiac, hepatic, or renal diseases, were excluded. Furthermore, women with limb deformities and disorders of nervous and musculoskeletal systems were also excluded from the study.

### Measured variables

The following anthropometric variables were measured in all subjects adhering to standard protocols [17]. Body weight was measured to the nearest 0.1 kg using a digital weighing scale (NAGATA, Tainan, Taiwan) while the subjects were wearing light clothes. The weighing scale is calibrated annually with daily spot check according to the manufacturer’s guidelines. Standing height was measured without footwear and recorded to the nearest 0.1 cm with a stadiometer (NAGATA, Tainan, Taiwan). Circumferences at three sites, right mid-upper arm, right mid-thigh, and right medial calf, were obtained in triplicate to the nearest 0.1 cm while subjects were standing erect. SFTs were measured in triplicate over the triceps, calf, and thigh on the right side using a skinfold caliper (Holtan Ltd., UK) to the nearest 1.0 mm. Measurement consistency between the consecutive measurements was considered as 1.0 mm, and if not, another separate measurement was taken. Three measurements that were within the acceptable range were then averaged. Details of the sites measured are given in Table 1. All the measurements were obtained by a single trained investigator to ensure the consistency of the measurements. The limb circumferences (*C*_limb_) were corrected for subcutaneous adipose tissue thickness [9, 10]. The corrected muscle (including the bone) circumferences (*C*_m_) were calculated as *C*_m_ = *C*_limb_ − π*S* (*S*, SFT). *C*_m_ was considered as corrected girth (CAG—corrected arm girth, CTG—corrected thigh girth, CCG—corrected calf girth). In addition, as appendicular circumferences are uni-dimensional and muscle mass is three-dimensional, *C*_m_ was squared and multiplied by height to convert to a three-dimensional measure [9, 10].

**Table 1:** SFT and circumference measurement sites

Site	SFT measurement	Circumference measurement
Upper arm	Triceps—measured in the midline posteriorly over the triceps muscle at a point midway between the lateral projection of the acromion process of the scapula and the inferior margin of the olecranon process of the ulna.	Measured midway between the lateral projection of the acromion process of the scapula and the inferior margin of the olecranon process of the ulna
Thigh	Measured at the midline of the anterior aspect of the thigh, midway between the inguinal crease and the proximal boarder of the patella	Measured midway between the midpoint of the inguinal crease and the proximal border of the patella
Calf	Measured on the medial aspect of the calf at the same level as the calf circumference	Measured at the maximal circumference

ASMM measured by DXA (ASMM_DXA_) (Hologic Discovery W, Hologic Inc., Bedford, MA, USA) was used as the reference standard in this study. Subjects were scanned in light, metal-free clothing while lying flat on the table. The same technician who calibrated the machine daily performed all scans and analyzed them according to the manufacturer’s guidelines to avoid inter-operator variability. ASMM_DXA_ was determined by the sum of SMM of lower and upper limbs [4]. Body fat percentage (%) was also measured with DXA.

### Anthropometric models validated in the study

The models considered in this study are explained in Table 2. The two models developed by Lee et al. [12] (ASM1, ASM2) and the two models developed by Wen et al. [16] (ASM3, ASM4) were considered for cross validation. The models ASM1 and ASM3 are based on circumferences and SFTs while the models ASM2 and ASM4 are based on body weight and height. Gender, age, and ethnicity are independent inputs in all four models. Table 2 presents information related to the original validation of the four models selected for this study.

**Table 2:** Models tested with the validation criteria and methods used by the authors

Models developed by	Criterion method used by authors	Models specification	Model	*R* ^2^	SEE
Lee et al. (2000)	MRI	Circumference-skin fold model	ASM 1 = height (0.00744 × CAG^2^ + 0.00088 × CTG^2^ + 0.00441 × CCG^2^) + 2.4 × sex − 0.048 × age + race + 7.8 (where sex: females = 0, males = 1 and race: Asian = − 2, African American = 1.1, Hispanics = 0)	0.91	2.2
		Weight-height model	ASM 2 = 0.244 × weight + 7.80 × height + 6.6 × sex − 0.098 × age + race − 3.3 (where sex: female = 0, male = 1 and race: Asian = − 1.2, African American = 1.4, Hispanic = 0)	0.86	2.8
Wen et al. (2011)	DXA	Circumference-skin fold model	ASM 3 = height (0.001509 × CAG^2^ + 0.000855 × CTG^2^ + 0.0007709 × CCG^2^) − 4.044 × sex+ 0.149 × weight − 0.038 × age + 12.246 (where sex: female = 2, male = 1)	0.92	1.44
		Weight-height model	ASM 4 = 0.193 × weight + 0.107 × height − 4.175 × gender − 0.037 × age − 2.631 (where sex: female = 2, male = 1)	0.90	1.63

*r* correlation coefficients, *R*^2^ determination coefficients, *SEE* standard error of estimate

### Statistical analysis

Data were analyzed using SPSS 20.0 (IBM statistics, Inc., Chicago). Descriptive statistics means (SD) or frequencies (%) were used to describe the data. Pearson correlation coefficients, unadjusted and then adjusted for age and menopausal status, were used to identify the associations between anthropometric variables and ASMM_DXA_. *P* value < 0.05 was considered statistically significant.

Pearson correlation coefficient (*r*) was used to determine the correlation between ASMM predicted by existing anthropometric models and ASMM_DXA_. The paired sample *t* test was applied to verify the differences between ASMM_DXA_ and ASMM predicted by models. Additionally, *R*^2^ and SEE were determined with regression analysis. The models were considered valid when there was no significant difference between the mean values, SEE was < 3.5 kg, and *R*^2^ was > 0.7 as recommended by Lohman [18] and later followed by Pereira et al. [13]. The models which fulfilled all three cross-validation criteria were further tested for repeatability with Bland and Altman plots [19].

### Ethical considerations

Ethical clearance for the study was obtained from the Ethical Review Committee, Faculty of Medicine, University of Ruhuna, Sri Lanka, and informed written consent was obtained from each participant prior to the commencement of the study.

## Results

The basic characteristics and anthropometric indices of study participants are shown in Table 3. Mean (SD) ASMM_DXA_ was 15.39 (2.75) kg. All anthropometric indices studied showed positive correlations with ASMM_DXA_ (Table 3). The results did not change materially when the correlations were adjusted for age and menopausal status.

**Table 3:** Physical characteristics of the study participants and correlation of anthropometric indices with ASMM_DXA_ (*n* = 165)

Characteristics/parameter	Mean (SD)	Correlation with ASMM_DXA_			
		Pearson correlation**	Age-adjusted partial correlation**	Menopausal status-adjusted partial correlation**	Age and menopausal-adjusted partial correlation**
Age (years)	49.1 (8.2)	–	–	–	–
Height (m)	1.50 (0.06)	0.55	0.53	0.53	0.53
Weight (kg)	57.52 (10.62)	0.87	0.88	0.89	0.89
BMI (kg/m^2^)	25.36 (4.32)	0.67	0.70	0.70	0.71
SFT at triceps (mm)	19.47 (6.09)	0.53	0.51	0.52	0.51
SFT at thigh (mm)	27.71 (9.90)	0.38	0.36	0.37	0.36
SFT at calf (mm)	18.63 (8.41)	0.43	0.41	0.41	0.41
Upper arm circumference (cm)	31.15 (3.85)	0.73	0.74	0.75	0.75
Thigh circumference (cm)	49.07 (7.41)	0.56	0.56	0.56	0.56
Calf circumference (cm)	34.40 (5.68)	0.52	0.51	0.51	0.50
CAG	25.03 (2.84)	0.60	0.63	0.62	0.63
CTG	40.35 (6.78)	0.47	0.47	0.46	0.47
CCG	28.54 (4.93)	0.25	0.22	0.23	0.22
CAG^2^ height	957.39 (229.21)	0.68	0.69	0.69	0.70
CTG^2^ height	2525.41(758.69)	0.59	0.59	0.59	0.59
CCG^2^ height	1264.75 (513.97)	0.25	0.23	0.23	0.23
ASMM_DXA_	15.39 (2.75)	–	–	–	–
Body fat percentage (%)	36.21 (5.53)	–	–	–	–

*BMI* body mass index, *SFT* skin fold thickness, *CAG* corrected arm girth, *CTG* corrected thigh girth, *CCG* corrected calf girth, *ASMM*_*DXA*_ DXA measured appendicular skeletal muscle mass

**All the variables were significantly correlated at < 0.001 level

Studied women had wider BMI (mean ± SD, 25.36 ± 4.32; range, 15.91–36.94 kg/m^2^) and body fat percentage (mean ± SD, 36.21 ± 5.53; range, 20.20–52.10%) ranges (Table 3). The correlations between body fat percentage and SFT of the triceps, thigh, and calf were *r* = 0.60, *r* = 0.60, and *r* = 0.55, respectively, and the correlations between body fat percentage and circumferences of the upper arm, thigh, and calf were *r* = 0.55, *r* = 0.39, and *r* = 0.34, respectively (data not shown in Tables).

Table 4 shows the results of cross validation of four tested models: ASMM estimated by these models showed strong correlations ranged between 0.68 and 0.82. ASMM estimated by ASM1 showed lesser *R*^2^ (46%) compared to the ASMM estimated by the other three models. ASMM_DXA_ and ASMM estimated with ASM3 were not significantly different (mean difference, 0.05; range of difference, − 0.23 to 0.12; *P* = 0.57); and ASMMs estimated by the other three models were significantly different from ASMM_DXA_ (Table 4).

**Table 4:** Cross validation of ASMM estimated with models developed by Lee et al. and Wen et al. with ASMM_DXA_ (*n* = 165)

Models		Pearson correlation and regression analysis			Mean (SD) (kg)	Paired sample *t* test			
		*r*	*R* ^2^	SEE (kg)		MD/SE (kg)	SD	Range of mean difference (kg)	*P* value
Lee et al. (2000)	ASM1	0.68*	0.46	2.02	18.36 (3.27)	− 2.97	2.45	− 3.35 to 2.59	< 0.001
	ASM2	0.90*	0.81	1.18	16.46 (3.01)	− 1.07	1.28	− 1.27 to − 0.87	< 0.001
Wen et al. (2011)	ASM3	0.90*	0.81	1.17	15.44 (2.40)	− 0.05	1.17	− 0.23 to 0.12	0.57
	ASM4	0.91*	0.82	1.14	14.44 (2.45)	0.94	1.14	0.76 to 1.11	< 0.001

*ASMM*_*DXA*_ ASMM measured with DXA, *r* Pearson correlation, *R*^*2*^ determination coefficient, *SEE* standard error of estimate, *MD* mean difference, *SE* standard error

*Correlations were significant at < 0.001 level

**Fig. 1:**
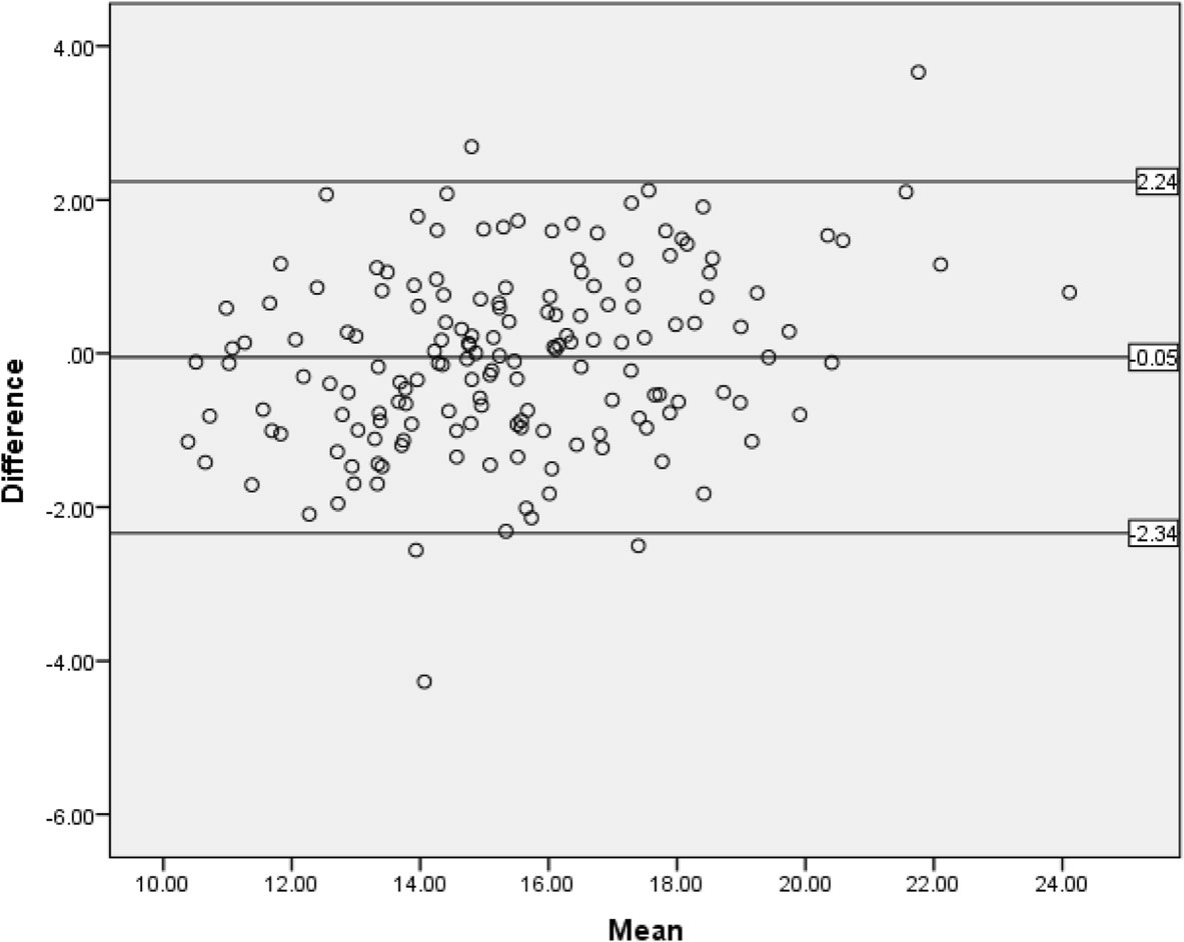
Agreement between ASM3 developed by Wen et al and the reference standard of ASMM (ASMM_DXA_) (n=165)

When the measurement agreement of the ASMM_DXA_ and ASMM estimated by ASM3 was tested by the Bland and Altman plot, more than 95% of values were within the limits of agreement (± 1.96 SD of the mean difference, − 2.24 to 2.34) indicating the accuracy of the ASM3 model (Fig. 1).

## Discussion

In this study, ASMM_DXA_ showed varying but strong correlations with ASMMs estimated by all selected models. ASM1 showed the highest measurement error while the other three models showed very similar results. ASM3, the circumference-skinfold model developed by Wen et al. [16], however, met all the validation criteria published by Lohman [18] and emerged as the best prediction model for this group of women.

Our data are consistent with Wen et al. [16] who have shown that the circumference-skinfold model (ASM3) is better compared to the weight-height model (ASM4) even though ASM4 is more practical and easily adoptable in any setting.

ASM1 and ASM2 [12] that have been developed using data from multi-ethnic US population did not meet the validation criteria we followed. These models have used data from non-obese males and females in a broad age range of 18–80 years and MRI as the reference standard. The current study included only women in a narrow age range, ages between 30 and 60 years, in wider BMI and body fat percentage ranges, and these may have accounted for the discrepancy we observed. This inconsistency is further explained by the lower values of height-adjusted ASMM in our women compared to NHANSE reference standards, measured using Hologic DXA [20]. Furthermore, we used DXA as the reference standard in our analyses.

Wen et al. [16] have also used subjects in a broad age range (19–69 years). However, the inclusion and exclusion criteria were similar to the current study. Further, they used DXA as the reference standard. The comparability of study samples may explain the measurement agreement shown by these models.

In adopting a model developed elsewhere to a local population, the disparities of the two populations with regard to the genetics, nutrition, and physical activities may play a significant role in determining the adoptability of such a model. This may partly explain the accuracy of ASM3 which is based on Chinese data, when applied to Sri Lankan subjects.

Development of country-specific prediction models is a daunting task, and many have attempted adopting models developed elsewhere to local population with varying success. Rech et al. [21] found a high agreement between measured and estimated ASMM in Brazil. A similar attempt has been made by Lekamwasam and Nanayakkara [22] who validated a weight- and height-based model in a group of Sri Lankan women.

In contrast to the previous study done in Sri Lanka, we found the model which included circumference and SFT (ASM3) to have better accuracy in estimating ASMM. We believe that circumference and SFT add new dimensions to the prediction model to improve its output. SFT is basically a measurement of fat mass, and circumference usually measures both SMM and fat mass. We observed that the relationships between body fat percentage and SFT of the triceps, thigh, and calf were stronger than the relationships observed between ASMM and SFTs. In contrast, the relationships between body fat percentage and circumferences of the upper arm, thigh, and calf were weaker than the relationships observed between ASMM and circumference. However, both menopause- and age-related changes in fat deposition and loss of skin elasticity contribute to variations in both measurements [23] and such adjustments used in these studies may overcome the errors in estimation of muscle mass.

In our analysis, the ASM3 is the best model to predict muscle mass and it would explain 81% (*R*^2^ = 0.81) of the muscle mass variation. One may consider adding other anthropometry measures such as bicep SFT and arm length to improve the predictability of these models. Further, utilization of site-specific anthropometry indices would provide more accurate prediction of muscle mass of specific regions. Therefore, upper arm circumference for the upper limb muscle mass and thigh circumference for the lower limb muscle mass [24] would be more appropriate than the current approach. However, the lack of facilities restricts the applicability of such an advanced method in the current study.

The current study has a few limitations. It involved only a sample of relatively healthy females aged 30–60 years selected from a single area of the country. This may limit the generalizability of the models, particularly in clinical situations. Prospective large-scale validation studies are required to determine the validity of these prediction models among different age groups and in different clinical situations.

## Conclusions

This study proved the ability of anthropometry-based models to estimate ASMM accurately in middle-aged women in our setting. Except the weight-height-based model by Lee et al., the other three models showed a high measurement concordance. Circumference skinfold-based model (ASM3) by Wen et al., however, satisfied all the criteria we followed and emerged as the most accurate model to estimate the ASMM in middle-aged Sri Lankan women.

## Abbreviations

ASMM: Appendicular skeletal muscle mass
ASMM_DXA_: Appendicular skeletal muscle mass measured by DXA
BMI: Body mass index
CAG: Corrected arm girth
CCG: Corrected calf girth
CT: Computer tomography
CTG: Corrected thigh girth
DXA: Dual energy x-ray absorptiometry
HRT: Hormone replacement therapy
MRI: Magnetic resonance imaging
NCD: None communicable diseases
PCOS: Polycystic ovarian syndrome
QOL: Quality of life
SD: Standard deviation
SEE: Standard error of estimate
SFT: Skinfold thickness
SPSS: Statistical package for social sciences
UK: United Kingdom
US: United States
USA: United States of America
